# Optimizing Gastrointestinal Integrity in Poultry: The Role of Nutrients and Feed Additives

**DOI:** 10.3389/fvets.2018.00348

**Published:** 2019-01-31

**Authors:** Sunday A. Adedokun, Opeyemi C. Olojede

**Affiliations:** Department of Animal and Food Sciences, University of Kentucky, Lexington, KY, United States

**Keywords:** exogenous enzyme, gut health, integrity, nonruminant animal, nutrition

## Abstract

Immunomodulation of the immune system by stimulating or suppressing one or both arms, is an emerging concept driven by the understanding of the host defense system. In particular, the gastrointestinal tract (GIT) functions not only as a site for digestion and absorption of nutrients but also acts as a metabolic and immunological organ. This serves as a barrier against abnormal presentation of luminal constituents, caused by dysfunctional intestinal epithelial barrier, to the mucosal immune system. Invasion by pathogens in the case of disease or stress or a massive influx of commensal bacteria overcomes the defensive mechanisms, resulting in the full activation of local dendritic cells and the expression of co-stimulatory molecules and pro-inflammatory cytokines. A growing body of literature demonstrates the immune benefits of increasing the intake of specific nutrients. This strategy involves formulating diets that encompass the bioavailability and utilization of nutrients from various food sources and understanding the dynamics of the macro and micronutrients to support all physiological functions as well as maintaining the function of the immune cells. The nature and type of feed ingredients may also play some roles on the integrity of the GIT of birds. Because dietary intake or nutritional status as well as nutrient requirements may be altered as a result of disease or stress, this may eventually alter the gut microflora and intestinal mucosal integrity, resulting in a compromised barrier of the intestinal epithelium. The weakening of the intestinal integrity could result in an increase in bacterial adherence to the mucosa, bacterial translocation, susceptibility to opportunistic bacterial infection, and mis-appropriation of nutrients. In this chapter, we will discuss the role of dietary energy and nutrients as substrates that have the potential to influence GIT's health and integrity and their roles, directly or indirectly, in modulating bird's ability to be resilient or resist infection.

## Introduction

Being the continuation of the external environment, the function of the gastrointestinal tract (GIT) of a bird, like any other livestock, includes protection against insults (infectious and non-infectious), transport of ingested feed and digesta along the GIT, digestion and absorption of nutrients and energy, secretion of endogenous materials, hosting of intestinal microbiota, and excretion of undigested portion of the ingested feed and metabolic waste (1). A healthy GIT will be able to efficiently carry out these functions while a compromised GIT may be unable to perform one or more of these functions. Although the integrity of the GIT of a bird depends on several factors, nutrients from diet play an important role in the maintenance of the integrity of the intestinal mucosa and gut microbial population (2–4). The timing of the first feed (early placement of feed), the quality (composition and physical texture) of the feed, as well as the quantity of the diet at an early age could influence the integrity of the GIT of the bird for several weeks (1, 5). In addition to the diet, efforts must be made to eradicate or minimize factors that could weaken or destroy the integrity of the GIT. Infectious agents such as bacteria (*Escherichia coli, Salmonella typhimurium* (6)*, Clostridium perfringens* (7)*, Campylobacter* etc.), intestinal parasites such as protozoan (e.g., *Eimeria species*) (8) and worms (e.g., *Ascaridia galli*), as well as stress arising from poor management (lack of adequate diets and/or water as well as sub-optimal barn or cage temperature) could compromise the integrity of the GIT (9). Toxins from mycotoxins found in feed ingredients have also been shown to be capable of negatively impacting intestinal integrity, reduce performance, and in some cases lead to high mortality (1, 10, 11).

The increasing growth of the world population and its food economy has resulted in a shift in diet and food consumption patterns toward animal products. Available data indicated that the poultry industry assumes a significant proportion of this increase in animal protein production and consumption (12, 13) which is characterized by a global increase in the production and consumption of poultry meat compared to other livestock products. Accompanying this growth, the poultry industry is faced with an enormous challenge to maintain the health and well-being of the birds. For several decades, the use of antimicrobial growth promoters (AGPs) and anticoccidia drugs became an integral part of the growing poultry industry. It was first used in non-ruminant animals' diet around the1940s (14). Antimicrobial growth promoters have been used either prophylactically to prevent an infection, therapeutically to treat an infection or sub-therapeutically as a growth promoter. According to the Center for European Agricultural Studies (15), a review of published studies from 1980 to 1989 showed increased growth performance (about 4%) and improved feed efficiency (5%) associated with the sub-therapeutic effects of AGPs. This gives antibiotics' use in livestock production an economic and health advantage. Observations from early studies on the mode of action of the growth-promoting effects of AGPs suggested that there is an interplay between AGPs and the gut microbiota (16–18). Coates et al. (16) observed that by adding AGPs to conventionally raised chick diet, body weight increased, and gut weight adjusted to constant body-weight decreased, with an apparent thinning of the gut wall compared to that of birds on the control diet. However, in the germ-free chicks, the growth-promoting effect of antibiotics was inconspicuous. Thus, several working hypotheses of the growth-promoting effects of AGPs have been governed by its ability to decrease competition for nutrients within the microflora and a subsequent decrease in growth-depressing microbial metabolites. Secondly, a thinner intestinal wall (reduced gut size or thickness) is often associated with a loss of mucosa cell proliferation during microbial fermentation, resulting in enhanced nutrient digestibility as well as a decrease in the proportion of nutrients required for gut maintenance (19, 20). However, because of the sub-therapeutic levels of AGPs administered to farm animals (at doses less than the minimum inhibitory concentration for most pathogens) and the diverse gut microbiota across various animal species, another plausible explanation has been contemplated. According to van den Broek (21), an interaction between phagocytes, microorganisms and the antibiotics cannot be overlooked. This is evident in how it exerts different inhibitory functions on inflammatory cells, chemotaxis and granuloma formation, the production of reactive oxygen species (ROS), and proinflammatory cytokine production (21–24). In this context, decreasing immunologic stress in the gut, through anti-inflammatory and immunomodulatory properties, AGPs inhibit sub-clinical infections before animals become overtly ill reducing the metabolic cost to the innate immune system (24). In view of this, unraveling the mechanisms through which AGPs improve livestock health and performance, lies in our ability to be able to piece together the role of the different activities occurring simultaneously and directing the host immune responses to interact with the intestinal microbiota. While modern-day livestock has benefitted from the use of AGPs and anti-coccidia drugs, a conundrum still exists. The re-emergence of “superbugs” that are resistant to chemotherapeutic treatment, poses a threat to public health. A widespread concern of AGPs overuse in livestock farming has resulted in its restriction and complete ban (in some cases) in livestock feed and this has led to a pressing need for an alternative to AGPs. This is important when evaluated from the welfare of the animal as well as the health implications for the consumers. The focus of this chapter is to examine the role of dietary energy, amino acids, micronutrients, and some feed additives in ameliorating the detrimental effects of stress to the bird's GIT.

## Bacteria

The most common challenge that the GIT of a bird faces is bacterial infections. In poultry, infections from *Escherichia coli, Salmonella typhimurium*, and *Clostridium perfringens* are some of the most common pathogenic bacteria that are associated with poultry production. The severity of bacteria disease will depend on factors such as the age of the bird and the load of the pathogen to which the bird is exposed to (feed, water, or the environment). This could be low grade with minimal damage to the intestine and minimal economic losses. However, in some cases, a bacterial infection could lead to significant economic loss as a result of sick birds and high mortality as seen in birds under severe necrotic enteritis (25, 26). This challenge has been effectively reduced with the inclusion of a sub-therapeutic level of AGP in the diets of poultry. However, due to concern relating to potential resistance to antibiotics (27) as well as consumers' preference, the use of AGP in poultry production is no longer desirable. Hence, there is the need to identify a new product, which must be natural (or organic) to replace AGP in birds' diet.

## Protozoan

In addition to the destruction or reduction in the integrity of the GIT as a result of bacterial infection, the role of intestinal protozoan, of the genus *Eimeri*a, which causes coccidiosis, has been shown to have the capacity to negatively affect the integrity of the GIT of poultry. *Eimeria* species are obligate intracellular parasites that exhibit a complex life cycle with developmental stages alternating between the external environment and intracellularly within the host (28, 29). While their virulence and pathogenicities differ among species, they cause moderate to severe intestinal lesions and induce both humoral and cell-mediated immune response. Although the incidence of *Eimeria* sp. have been drastically reduced with appropriate vaccination and the use of anti-coccidia drugs in the diets of poultry however, huge economic losses (more than US$3 billion worldwide), is still being incurred annually (26, 30). In addition to mortality that may arise from these parasites, a significant economic loss from morbidity [as a result of a reduction in feed intake, nutrient, and energy digestibility, and performance; (31–34)], the destruction of the villi and crypt (shorter and thicker villi), and a reduction in tight junction functionality have been reported. Birds are infected when the oocytes of the protozoan are ingested through water, feed, or from the litter on which they are raised. The oocysts hatch within the GIT within a few days and by day 5–7, the effects of these parasites on the bird's performance reaches its peak as revealed with a significant reduction in feed intake, oocyte shedding, and body weight gain. These parasites cause tissue damage which typically results in partial or complete destruction of villi and intestinal mucosa. Indeed, *Eimeria* sp. infection usually opens the door to secondary infections such as necrotic enteritis caused by *Clostridium perfringens*. In addition to vaccination against coccidiosis administered on the day of hatch, anti-coccidia drugs are added to the diets to prevent coccidiosis, however, with the current trend of increasing demand for organic poultry products, the use of anti-coccidia drugs in poultry diets may soon be completely phased out. By tapping into novel concepts to mitigate the effects of *Eimeria* on gut health and function, Kim et al. (35) tested the effects of epidermal growth factors (EGF) on gastrointestinal health. Epidermal growth factor, a ubiquitous polypeptide, is said to be capable of stimulating the proliferation and differentiation of epithelial cells. While EGF did not improve growth performance, they observed an improved expression of genes for nutrient transporters and tight junction proteins in *Eimeria* challenged birds (35), suggesting a cellular proliferation and rejuvenation of intestinal cells to replace damaged enterocytes during infection and inflammation. Application of molecular methods (genomics and proteomics) to provide mechanistic information on stress-induced underpinning lesions, produced in the GIT will be important in defining the role of growth factors, inflammatory cytokines, and regulatory factors in cellular proliferation, morphogenesis and tissue repair of intestinal integrity.

## Worms

Consumers of poultry products have enjoyed the supplies of healthy and wholesome meat and eggs for several decades. This is as a result of adequate veterinary care through careful use of appropriate medications to prevent or treat poultry disease(s). With an increase in demand for poultry products, especially eggs, from birds that are raised “naturally” by the consumer (including organic products), birds are increasingly being raised outdoors on pastures. This situation has led to an increase in the incidence of intestinal parasitic infections, especially by GIT helminths including nematodes and cestodes. These incidences are common in laying hens compared to broilers and this could be explained by the fact that laying hens live much longer that broiler chickens, hence they are at a higher risk of worm infection. With increasing number of organic farms being operated to cater for consumers' need for eggs from organic flocks of laying hen, the prevalence of *Ascaridia galli* is likely to increase (36). In addition to the high risk of contamination of poultry products (meat and eggs) with eggs from parasitic worms, the possibility of some of these worms finding their way into poultry eggs exists. Although there are currently data on some natural products [bioactive compound from herbs, botanicals, essential oils, and oleoresins (37)], it is extremely difficult to replicate the results from some of these studies due to factors that include experimental design, lack of enough methodological details, how the worms and eggs were treated prior to being used in the study etc. as discussed in the later part of this chapter. Additionally, the difficulty of extraction of the bioactive compound from these potential products as well as information on dose recommendation for optimal use in poultry is scarce and inconsistent.

## Stress

Stress is another factor that could predispose poultry to enteric disease including leaky gut and GIT enteritis. Stress could be caused by several factors including environmental (sub-optimal temperature), dietary (feed deprivation, unbalanced diet, sub-optimal feed, and ingredient quality, etc.), vaccination- and medication-induced stress, microflora imbalance induced stress, as well as stress as a result of pathogen or parasitic load (bacteria, protozoan, or intestinal worms). Although almost all of these could reduce the protective capability of the gut, the mechanism through which this occurs are different and hence, would require different approaches in evaluating the efficacy of an alternative product to combat this challenge. Stress to the normal functioning of the GIT will result in the disruption of the balance between the production and elimination of the ROS (38). The high level of ROS in the intestinal cells will result in the destruction of the polyunsaturated fatty acids in the membrane of cells leading to the production of peroxides which could eventually lead to the production of malondialdehyde (MDA) which has been implicated in the gradual destruction of the integrity of the cell membrane. The effect of MDA on the integrity of the GIT cell membrane includes nutrient malabsorption, morbidity, or mortality. A compromised intestinal epithelium creates a good opportunity for opportunistic pathogens to cause an infection. In addition to this, dietary deficiencies in certain nutrients can increase the stress-induced susceptibility of poultry to oxidative stress (38–40). Loss of intestinal integrity and functionality will lead to malabsorption, a decrease in performance, bacterial translocation, product (meat and egg) contamination, morbidity, and in some cases death. The outcome of this is an economic loss to the producer.

## Gut Microbiome And Associated Immune System

From birth to death, mucosal surface (including the skin, the GIT, etc.) of virtually all vertebrates are colonized by a vast array of complex and dynamic populations of microorganisms. Nobel laureate Joshua Lederberg suggested using the term “microbiome” when describing the collective genome of indigenous microbes (microflora) in the GIT (41). This microflora is composed mostly of bacteria, and to different degrees archaea, viruses, fungi, and protozoa. The GIT harbors the largest population of these organisms with over 640 different species of bacteria and more than 20 different hormones (42). These are continuously exposed to different antigens, which can be either pathogenic or nonpathogenic such as foods and commensal organisms. The list of beneficial functions attributed to intestinal bacteria continues to grow and includes nutrient processing, regulation of intestinal angiogenesis, development of gut-associated lymphoid tissue (GALT), induction of oral tolerance, mucosal immunity, and diversification of the pre-immune antibody repertoire (43). The relationship between the intestinal microbiota and the host is tightly regulated and reflects co-evolution among the inhabiting microbes, genetic, immune, and metabolic interactions with the host, and environmental influences (44). Although, the mechanisms that maintain intestinal homeostasis are just now becoming clear, evidence particularly from studies of rodents and humans has enabled the unraveling of the balance that exists between the host and its microbiota. According to Hooper and Gordon (41), these interactions can be viewed in terms of a continuum between symbiosis, commensalism, and pathogenicity. In this case, there is a fine line in the relationship between the host and microorganism from when it becomes beneficial, neutral, or detrimental to the host. This is evident in cases of intestinal epithelium damage where an opportunistic invasion of host tissue by resident bacteria can pose a serious health consequence including inflammation and sepsis. Accordingly, GALT develops in a manner that allows non-pathogenic substances, such as commensal bacteria, to survive and enables tolerance to food antigens while protecting the host from pathogenic organisms and other potentially toxic substances.

One of the main characteristics of the gut is to be sufficiently permeable to support efficient absorption of nutrients, it must avoid potentially damaging immune responses to dietary proteins and commensals. This dynamic and reciprocal interactions between the microflora, intestinal epithelium, and the immune system can be targeted to improve gut health. We know that the immune system has evolved adaptations that work together to contain the microbiota and preserve the symbiotic relationship between host and microbiota, ultimately protecting the host from pathogens and fostering complex microbial communities for their metabolic benefits. The tissues of the GIT are rich in myeloid and lymphoid cells, many of which reside in organized lymphoid tissues. The GALT is a key immunological system estimated to comprise more immune cells than any other tissue (45) with the associated structures forming a site to promote co-localization of the many immune cell types required to initiate and mediate immune function. Many of the organized GALT structures are sites of immune induction (46, 47) providing conditions necessary to induce appropriate immune responses (e.g., immunoglobulin IgA production by plasma cells). There is also considerable cellular traffic between different gut immune structures and the systemic sites including the bone marrow and spleen. Thus, the gut microbiota directs maturation of the host immune system, by eliciting antigen-specific responses which are taken up by resident dendritic cells. However, because these microbes are non-invasive, resident phagocytes are not fully activated but they stimulate a finely balanced response inducing the production of IgA which controls host-commensal interaction by both impacting commensal gene expression in the lumen and preventing adhesion of commensal bacteria to the epithelial surfaces. In the case of an infection or exposure to any variant of stress. Klasing (48) suggested that an animal susceptibility is dependent on its resistant and resilience capacity. Resistance is described as the ability to limit pathogen burden while resilience is the ability to limit the health impact caused by a given pathogen burden by maintaining productivity (e.g., growth, feed efficiency, egg production). In this context, not only do we need to be familiar with the mechanisms that are used to kill pathogens and prevent infection, a systemic understanding of how the body regulates the production, repair, and avoidance of the damage accumulated during an infection becomes imperative.

Defense against an infectious challenge requires a highly orchestrated response by the immune system. This is especially true for animals and birds that live in an environment with high pathogen load. A specific response against infection by potential pathogens, such as the production of antibodies against a particular pathogen or ROS during an immune response can be costly to the animal. By diverting the expenditures of energy and resources to the immune system surveillance, the overall performance of the bird is negatively affected. However, increasing the resilience of the animal by intentional manipulation through diet even before the occurrence of an infection, will not only confer protection but will also be advantageous in terms of productivity. Today, an interplay between diet and modulation of the immune system is a major topic of interest both in humans and livestock most of which addresses the possible maintenance or enhancement of gut health which has led to several practical applications.

## The Nexus Between Nutrition And Gastrointestinal Health

Diet and lifestyle are crucial factors that influence the susceptibility of humans to metabolic diseases. While this can be somewhat true for livestock, the diets we provide to our birds are geared toward meeting their nutritional needs without compromising any of the desired production characteristics. The question that arises is, is the requirement set to maximize productivity in healthy birds optimal for immunocompetence and disease resistance? Using dietary protein intake as an example, at elevated temperatures, digestion and absorption are altered, favoring protein catabolism, and subsequently a reduction in protein synthesis and deposition (49). The ideal amino acid balance under high temperature remains unclear as several strategies have been invoked. Alleman and Leclerq (50) reported that low protein diets (20 vs. 16 %) impaired broiler performance at high temperature (32°C) from 21 to 42 days of age while Temim et al. (51) reported that high protein diets (28 and 33 %) compared to low protein diets (20%) slightly improved chick performance. Burkholder et al. (6) observed changes in commensal intestinal microbial populations evident by the attachment of *Salmonella enteritidis* to the ileal tissue, which increased when birds were either fasted for 24 h or exposed to high temperature (30°C) compared to the controls on the same diet.

The relationship between nutrition and immune competence has been explored over the years and more importantly, how it influences the overall health of the animal. Increasing evidence emphasizes how the nutritional value of feed is influenced in part by the structure and operations of the gut microbiome, and how that feed, in turn, shapes the microbiota. Furthermore, the nexus between nutrient metabolism and immune system as described elegantly by Klasing (48) operates through several mechanisms. These include the development of immune cells and tissues necessary for synthesizing effector cells, proliferation of certain pathogens by modifying the population of microorganisms in the GIT, providing substrates for the production of cells and molecules such as leukocytes that respond to infectious challenges, indirectly activating the endocrine system, and strengthening the intestinal epithelium against pathogenic assault. Thus, assessing immunological parameters in relation to nutritional status becomes paramount. As alluded to previously, the limit of AGPs and other drugs in livestock farming drives the need for an alternative approach to maximize productivity and to control enteric pathogens and parasites previously contained using AGPs and anticoccidia drugs in feeds. To maintain an optimum gut health in our birds, it is important to take advantage of the beneficial effect of consuming certain nutrients, beyond what is normally supplied from the diet for optimal growth and productivity. A good understanding of the aspect of gut and immunity as they relate to the maintenance of a healthy GIT flora, the modulation of the body's natural defenses systems including resistance to specific infection, improvement in diet formulation strategies to promote efficient energy and nutrient utilization is essential in order to enhance and maintain the integrity of birds. Dietary strategies including the use of major nutrients like carbohydrates, proteins (amino acids), lipids, as well as vitamins and minerals, or feed additives such as feed enzymes, pro- and pre-biotics, and antioxidants are known to play important roles in nutritional immune responses.

### Dietary Strategy

#### Energy and Protein

Malnutrition and infection are major obstacles to survival, health, growth, and reproduction of animals and humans worldwide (52, 53). This global concern has led to the development of remarkable advances in immunology and nutrition in recent decades to shed light on the effect of various nutrients on specific GIT functions including immune response and how they influence host resistance to infection. One of the major causes of immunodeficiency globally has been attributed to protein and energy malnutrition (52, 54). The provision of diets to poultry that meets the requirements for energy and nutrient in the era of sub-therapeutic use of AGPs has been fully mastered. With partial to complete withdrawal of AGPs in the diet of swine and poultry, the ensuing challenge is how would this affect energy and nutrient utilization of poultry but more importantly, how would the nutrient requirements of poultry change in light of renewed insults on the GIT by bacteria and intestinal parasites. The former could easily be addressed while the later poses a huge challenge with the understanding that different poultry feeding operation and different species or strains of poultry experience unique challenges, and the age of the animal may also influence the severity of this challenge. Although, adequate levels of nutrients in the diets of animals play important roles in maintaining an “optimal” immune response, deficiencies, and in some cases, excessive intake, could have negative consequences on the immune status and susceptibility of the animals to a variety of pathogens. It has been shown that the strongest determinant of the gut microbial profile is the host's diet (2–4). Factors such as diet composition, nutrient density, diet physical characteristics, ingredients and diet processing method (feed processing techniques), and type of feed additives play significant roles in the dynamics of the GIT microflora. From a nutritional viewpoint, substrates (e.g., amino acids, energy, enzyme co-factors) are needed to support the clonal proliferation of antigen-driven lymphocytes, the recruitment of new monocytes and heterophils from bone marrow, the synthesis of effector molecules (e.g., immunoglobulins, nitric oxide, lysozyme, complement), and communication molecules (e.g., eicosanoids, cytokines). Recent studies indicate that dietary protein deficiency, which reduces the concentrations of most amino acids in plasma (55) and compromises the immune system, can suppress immune response by decreasing lymphocyte number, overall leukocyte count, and splenic cell proliferation stimulated with phytohemagglutinin-M (56–58). Moreover, both immune systems (innate and adaptive systems) are highly dependent upon an adequate availability of amino acids for the synthesis of these proteins and polypeptides, as well as other molecules with enormous biological importance (59). These substances include nitric oxide (NO), superoxide, hydrogen peroxide, histamine, glutathione, and anthranilic acid.

For nutritional purposes, amino acids have been divided into two groups; essential and non-essential dietary amino acids. Essential amino acids are those that cannot be synthesized endogenously, or at the rate that is sufficient to meet physiological needs (including maintenance, growth, and reproduction) of the bird and must be supplemented in the diet. Non-essential amino acids, on the other hand, are those that can be synthesized endogenously from a non-amino acid source. Approximately 90% of diets for poultry in the U.S. is comprised of corn and soybean meal. Because the cost of these key ingredients has increased markedly in recent years, keen interest exists in feeding reduced protein corn-soybean meal diets with an adequate level of supplemental crystalline amino acids. It is to be noted that metabolically, several of the amino acids defined as essential can be synthesized from precursors that are structurally similar to these amino acids. Amino acids are an important class of nutrient that is needed for gut health and the ability of the bird to fight infection. During immunological stress, a higher level of available amino acids (needed for growth) is repartitioned to produce cytokines (e.g., interleukin-1, interleukin-6, and tumor necrosis factor-α) which alter the overall protein metabolism. Based on this, a higher level of certain amino acids may be required in the diet of birds that are raised under a relatively higher pathogen load.

Individual amino acids affect immune responses either directly or indirectly through their metabolites. The role of these amino acids (glutamine, arginine, tryptophan, and cysteine) on the integrity, growth, and development of the intestinal epithelium, gene expression, cell signaling, antioxidative responses, and their associated immune functions have been investigated (8, 39, 55, 60). In particular, the role of glutamine, arginine, tryptophan, and cysteine (39, 55, 60) has been reported. Gao et al. (39) showed that *in ovo* feeding of arginine influenced the development of lymphoid organs in broiler chicks while, Tan et al. (8, 61) showed that L-arginine supplementation could regulate the immune function in challenged birds. In addition to this, Lee et al. (62) showed that arginine had a positive effect on the chicken cellular response to infectious bronchitis virus with its potential to function in the repair of damaged intestinal epithelium cells by activating the mTOR pathway (63). Glutamine, a precursor for several biosynthetic pathways, is required for growth and cell division and a principal metabolic fuel for enterocytes, lymphocytes, macrophages, and fibroblast (64, 65). Classified as a non-essential amino acid, its requirement may not be able to meet the optimum level for specific conditions such as stress, infection, or injury that birds may be predisposed to, due to extensive genetic selection (66). Dai et al. (67) reported a significant improvement in weight gain and feed efficiency in broiler chickens supplemented with glutamine. Improved meat quality and humoral immune response in poultry associated with better development of the intestinal mucosa have also been observed (68). Supplementing glutamine in the diet may be beneficial, not only in hyper-catabolic states but also in the maintenance of optimal health and maximal rates of growth in healthy animals (65). Glutamine is a good source of energy for mesenteric lymph nodes lymphocytes (59, 69) and is essential for the proliferation and function of lymphocytes (52). It enhances phagocytic activity of macrophages, and the production of cytokines and antibodies by T and B lymphocytes (59, 70), as well improving the growth of chicks (68). Threonine is another important amino acid that is abundant in the mucin that lines the entire GIT. An adequate dietary level of threonine has been shown to enhance intestinal integrity in poultry (4, 71).

In terms of quality of the different feed ingredients, it has been reported that animal protein sources, such as meat and bone meal, has the potential to enhance the proliferation of the bad bugs such as *Clostridium perfringens*, as a result of its high collagen and elastin contents that are resistant to endogenously secreted digestive enzymes in swine and poultry (72–74). Additionally, the nature of the starch crystallinity could delay amylase action on carbohydrate recovery (75–78). Proper selection coupled with an optimal level of inclusion in the diet is essential. Cereal (corn, wheat, sorghum, etc.), and legumes (soybean meal and canola meal) make up more than 80% of the diets given to poultry, all of which contain non-starch polysaccharides (NSP). Depending on the composition of the diet and the inclusion level, poultry, in general, lacks the ability to effectively break down the NSP in the midgut due to the shortage or the absence of substrate-specific endogenous enzymes capable of the breaking down NSPs, hence they exhibit a decreased nutrient digestion and absorption. An estimated 400–450 kcal of digestible energy per kg of feed remains undigested by broilers because of the NSP content present in corn-SBM diets (79). Hence, the use of NSP enzymes (carbohydrases) have been explored to ensure breakdown, degradation, and utilization of most of the components in corn and SBM to attain ideal performance and profit from these diets. This also minimizes the quantity of undigested NSP that reaches the hindgut, hence reduces the proliferation of harmful bacteria. High levels of NSP in these diets can also predispose the chickens to necrotic enteritis, a disease that has become prevalent with the removal of AGPs. The primary challenge is to minimize the exposure of birds to potentially damaging bugs as well as the insults arising from such a challenge on intestinal and mucosal integrity. This becomes paramount because, an increase in intestinal inflammation and ROS level, and a reduction in intestinal membrane integrity through a reduction in tight junction functionality is one of the signs of gastrointestinal infection in non-ruminant animals (8, 78, 80). The composition of the feed given to poultry is important. Diets that are deficient (quantitatively and qualitatively) in energy and nutrient have the tendency to limit the ability of the bird to be able to react accordingly to any developing insult in its GIT. Because of the tendency of the bird to reduce feed intake as a result of intestinal infection and inflammation, it may be necessary to increase the density of certain nutrients to equip the bird against any challenge (8, 81). Similarly, to minimize productive and economic losses as well as improving livestock welfare in the era of no AGP in the diets of non-ruminant animals, it is essential to look for a solution that is effective but also acceptable to the consumers. Based on consumer demand, the way to address this challenge may be the use of natural or organic products. Therefore, close attention should be placed on the quality of the different feed ingredients that goes into the diets of poultry. For example, feeding a highly digestible diet has an advantage over diets that contain relatively higher indigestible components such as NSP or resistant starch. The higher the quantity of the undigested portion of the diets that reaches the hindgut, the higher the probability of such promoting the growth of microbiota that may be potentially harmful to the integrity of the GIT of the bird. Early feeding is another dietary and management approach that can enhance and strengthen intestinal integrity. It has been suggested that the sooner the birds are exposed to nutrient-rich diet the better the development of the GIT mucosal. Thus, it is important to adequately provide the much-needed nutrients to birds especially during the transition period from *in ovo* nutrient utilization from the yolk to their gradual reliance on nutrients from the diet (82). What happens during this transition period could be critical to the health and integrity of the GIT of the bird later in life. The advantage of the *in ovo* feeding of the developing embryo during the late incubation stage could be taken.

#### Micronutrients and Feed Additives

In a different capacity, adequate levels of vitamins and minerals are essential for the birds to efficiently utilize dietary nutrients, post-absorption, for growth, health, reproduction, and survival. Most vitamins cannot be synthesized by poultry in sufficient amounts to meet physiological demands, hence must be obtained from the diet. Vitamins are present in many feedstuffs in minute quantities and can be absorbed from the diet during the digestive process. In general, chronically severe deficiencies of these micronutrients are more debilitating to the development of the immune system than macronutrients such as energy and protein. Nutrient deficiencies that are especially damaging to the development of the immune system include linoleic acid, vitamin A, iron, selenium, and several of the B-vitamins. Adequate levels of dietary selenium, nucleotides, long-chain polyunsaturated fatty acids, and vitamins A, C, and E in modulating the host defense against infectious pathogens have been reported (52). Vitamin A deficiency (83, 84) and excess (84, 85) have been shown to depress immune responses in chicks. Most research suggests that vitamin A deficiency is associated with reduced cellular immune responses whereas vitamin A excess impairs antibody responses. Vitamin E is primarily known for its role as an antioxidant in reducing cellular free radical damage, but its deficiency could lead to a reduction in immune responses (86). Male broilers fed diets varying in DL-α-tocopherol acetate from 0 to 87 mg/kg of diet exhibited altered thymic and splenic T cell populations indicating that more helper T cells (CD4) were present with increased dietary vitamin E and thus improved responsiveness to immunologic stimuli (87). Growth performance and immunity as affected by drinking water fortified with vitamins and electrolytes were evaluated in heat stressed broilers (88). The addition of B-vitamins, fat-soluble vitamins (A, D, and E), and electrolytes to drinking water improved aspects of antibody production to the serum red blood cell (SRBC) over that of control birds, and reduced broiler mortality from the heat stress. The trace minerals that have been associated with an improvement in immunity, or functions that support immunity, are Zn, Mn, Cu, and Se. Dietary Se interacts with vitamin E in antioxidant protection of cells because it is a component of glutathione peroxidase. Dietary Se intake increases TCR signal strength through mechanisms that involve free thiol concentrations. In addition to antioxidant status, Se has been shown to impact disease resistance. For example, broilers infected with *E. tenella* had improved resistance (i.e., reduced mortality and cecal lesions) when supplemented with Se (89). The immune system is dependent on the functions of cellular metabolism with Zn being central in cellular metabolism and functions both structurally and catalytically in important biochemical pathways. It has been hypothesized that the antimicrobial effect of Zn leads to growth promotion where gut microbiota is altered to reduce fermentation loss of nutrients and to suppress gut pathogens (90). Similarly, other evidence suggests that pathogens can have a competitive advantage over the commensal microbiota under Zn-limiting conditions, thereby being promoted under an inflamed state (91). Recently, it was shown (91) that Zn competition exists in *C. jejuni* and other bacterial species in the host microbiota of conventionally-raised vs. germ-free broiler chickens (*Gallus gallus*). Under conditions of Zn deficiency, preferential growth of bacteria able to survive at low-Zn levels might ensue. Furthermore, many recent studies have shown that prophylactic doses of Zn (as Zn oxide, ZnO) in various animal models increased the presence of Gram-negative facultative anaerobic bacterial groups, the colonic concentration of short chain fatty acids (SCFAs), as well as overall species richness and diversity. Adequate levels of Zn supplementation (between 50 and 70 mg/kg) in poultry diet have been shown to reduce the production and minimize the impact of oxidative damage in the intestine of broilers under intestinal stress (38, 40). This demonstrates the need for nutrient-directed management practices to reduce the effect of pathogens on the GIT of poultry (52). The level and nature of micronutrients in the diet given to poultry could be used to control the population and diversity of hindgut microbiota.

Furthermore, about 80% of the feed ingredients in poultry diets is plant-based, hence the use of exogenous enzymes such as phytase, which liberates phytate- and phytic-acid bound phosphorus (and other nutrients as a result of extra phosphoric effects), protease to enhance protein digestion, and carbohydrases for NSP breakdown is essential (92). One of the ways through which the NSP-digesting enzymes function is by reducing digesta viscosity which subsequently allows digestive enzymes to gain better access to the digesta and hence, increase nutrient and energy digestibility and absorption. Secondly, the passage rate of the digesta is slowed down allowing for sufficient time for digestion and absorption to take place. Carbohydrase enzymes indirectly could enhance GIT health by reducing the wetness of litter which could result in a reduction in the buildup of pathogenic organisms in the litter. The combination of these actions will lead to a reduction in the quantity and quality of nutrient and energy that reach the hindgut thereby denying pathogenic organisms the needed nutritional support needed for proliferation (78, 93). By supplementing the diet with enzymes, Jia et al. (94) observed an improvement in growth performance as well as a reduction in the negative effect of *Clostridium perfringens* on birds' performance. Their rationale was that NSP- degrading enzymes might reduce microbial activity because of substrate limitation in the ileum. Further evidence of their beneficial effect is the ability of NSP, through depolymerization, to generate galacto-, gluco-, or manno-oligomers, which can serve as prebiotics stimulating the growth and activity of lactic acid bacteria (95, 96). Another component of the diet of swine and poultry is the phytin which has been implicated in poor phosphorus digestibility in poultry. However, it has been reported that the breakdown of this structure in the gizzard could result in the formation of phytic acid that is negatively charged and could interfere with protein digestion [poor protein digestion (97–99)] in the diet which eventually becomes a rich source of nutrients to the microbiome in the hindgut. Although pro- and pre-biotics have been shown in some cases to show some potentials as alternatives to AGP, the lack of consistency in published data complicates this promise. Additionally, some of the inconsistencies or lack of significant effects could be explained, in part, by the nature of experimental design (appropriate design, and an adequate number of replicates), the age of the birds, and the availability of sufficient substrates for the enzymes.

In most cases, the inclusion of feed additives in poultry diets has resulted in improved feed intake and growth performance with a resultant improvement in feed efficiency. With the ban on AGP, phytogenics (a relatively new group of feed additives), have the potential to be embraced by the consumers as an alternative to AGP. Unlike drugs, these products are looked at as being of a natural origin. For more than a century, it has been recognized that certain plants, especially their secondary metabolites, have medicinal properties and have been used both in human and animal medicine with some products displaying antioxidative properties as well as other beneficial effects on the GIT (100–103). This group of compounds is large, and this makes their classification quite challenging and variable. Windisch and Kroismayr (37) have attempted to classify these plant products into four broad categories. These groups include herbs, botanicals, essential oils, and oleoresins. Herbs are produced from flowering and non-woody plants while botanicals are produced from roots, leaves, and bark of entire or processed plants (37). Essential oils, which is one of the most common group from this classification, are produced from hydro-distilled extracts of volatile plant compounds while oleoresins are extracts from non-aqueous solvents. Despite the potentials that these products possess, there is still a significant issue with purity, adequate description, and established dosage levels. Most of the available phytogenics have been shown in *in vitro* studies to possess antimicrobial and growth promoting effects, as well as being able to enhance the digestive process (103, 104).

## Looking Forward

Efforts should be placed on developing an alternative to AGP as well as materials for the control of intestinal parasites (worms and *Eimeria* sp.) that are acceptable to the consumers, in this case, “natural product.” The process of evaluating any alternative products must be cognizant of the welfare of the birds and the concerns of the consumers. With increasing number of birds raised on pasture, coupled with an increase in the level of interaction between birds (e.g., battery cage vs. aviary systems; intensive vs. semi-intensive production systems), there is the need to redefine biosecurity to take into account the latest development in how birds are currently being raised. In order to fully evaluate any product (e.g., as an alternative to APG), a lot of efforts must be placed on the design, the health status of the animals, and the products (type, dose, parameters to measure, and when such samples are to be collected).

## Methodological Considerations

In order to effectively evaluate the efficacy of a product such as an alternative to AGP, it is essential to create an environment in which the birds can respond to the treatments. For instance, AGP will not be as efficacious in a healthy bird that is raised in a clean or new poultry barn compared to a bird that is raised under conditions that naturally will predispose it to a certain type of stress or infection, in this case, intestinal stress, or infection. In order to achieve this, birds must be subjected to a certain level of intestinal stress and challenge. Moreover, the design of the study must include a positive control that is unchallenged as well as a challenged positive control+AGP. Furthermore, there should be a negative control (challenged without AGP) and the negative control treatment (diet) to which the product that is being evaluated would be added. Contamination of any kind (feed, bird, water, litter, cages, etc.) should be avoided and efforts must be made to determine, quantitatively, the concentration (or activity level) of the product that is being evaluated. Furthermore, it is essential to make sure that the positive and negative control diets are similar in energy and nutrient composition as much as possible in order to make sure that we do not inadvertently create different intestinal microbiota as a result of slight differences in the composition of the experimental diets. The tendency for the challenged birds to consume less feed has been widely reported; hence, depending on the study, it may be important to pair-feed the birds to the feed intake level similar to that of birds on the negative control diet (usually the treatment in which the birds consume the least amount of feed). This will control the feed intake and the quantity of the test product(s) that birds across all the treatments will consume. If this is carefully done, any interpretation of significant effect could be strongly attributed specifically to the test material. It is understandable that birds could be different from one location to the other; however, it is essential to include in the report as much information as possible. This would allow the reader to draw his or her own conclusion based on the information provided. Information such as the age of the bird, species or strain of the bird, gender, genetics, vaccination program, medication program (if used), room temperature and humidity, mortality, and morbidity should be provided. Proper experimental design with an adequate number of replications is essential for data analysis and interpretation. Finally, as the volume of research in the area of gut health in poultry and swine in response to withdrawal of AGP increases, it may be essential for researchers to develop some important biomarker of gastrointestinal functionality against which results from these studies may be standardized. Similar steps have been taken by nutritionists in the area of standardized ileal amino acid digestibility. According to Celi et al. (105), parameters to be measured to access the proper functioning of the gut should include, diet, effective digestion and absorption biomarkers, microbiota community, effective immune status, gut mucosa, and neuroendocrine and motor function of the gut. In line with these parameters, the development of biomarkers of gut health is imperative to gain clarity of understanding the pathophysiological events that influence the intestinal barrier, its functionality and the ecology of the GIT microbiota. Biomolecular monitoring of the GIT could offer rapid but precise disease detection and management mechanism by providing non-invasive strategies to define potential pathways behind the pathogenesis of diseases. Furthermore, this can assist in the assessment and diagnosis of various gastrointestinal conditions.

## Summary

Maintaining a healthy gut in our birds would continue to be a challenge for the foreseeable future. This will not be as a result of our inability to come up with products that will, to a reasonable extent, be able to fill in the gap left with the withdrawal of AGP, but rather coming up with a product that the consumers will readily accept. Based on the current trend, this product must be a “natural” product. If this trend continues, our ability to be able to accurately identify and extract products that are able to protect and enhance the development of the “good bug” and eliminate the “bad bug” will be crucial. To maintain the integrity of the GIT may require more than one product but a combination of products that could exhibit both pro- and pre-biotic effects on the GIT. Furthermore, future poultry breeding and selection program should include genes responsible for the bird's ability to resist an infection as well as the ability of the bird to be resilient in the face of high pathogen load. It is a common observation that with a group of birds that are fed the same diet, raised in the same space, and subjected to the same environmental conditions (similar level of stress, physical or biological), a few of these birds are able to completely resist an infection, some are able to cope with the infection (resilient), while others easily succumb to the infection. Evidently, in addition to the economic traits, the selection criteria should include those genes that make some of these birds resistant or resilient to gastrointestinal challenge. This means a holistic approach through novel strategies is necessary to minimize the impact of these stressors on poultry GIT health.

## Author Contributions

OO helped with research for some of the articles used when writing this article. OO wrote a portion of the manuscript (part of the introduction and a portion of the following section: GUT MICROBIOME AND ASSOCIATED IMMUNE SYSTEM) under direction from SA. SA wrote a significant portion of the article and reviewed and edited the manuscript before submission.

### Conflict of Interest Statement

The authors declare that the research was conducted in the absence of any commercial or financial relationships that could be construed as a potential conflict of interest.

## References

[1] Yegani M Korver DR. Factors affecting intestinal health in poultry. Poult Sci. (2008) 87:2052–63. 10.3382/ps.2008-00091 18809868 PMC7107194

[2] Pan D Yu Z. Intestinal microbiome of poultry and its interaction with host and diet. Gut Microbes (2014) 5:108–19. 10.4161/gmic.26945 24256702 PMC4049927

[3] Munyaka PM Nandha NK Kiarie E Nyachoti CM Khafpour E Impact of combined β-glucanase and xylanase enzymes on growth performance, nutrient utilization and gut microbiota in broiler chickens fed corn or wheat-based diets. Poult. Sci. (2016) 95:528–40. 10.3382/ps/pev333 26574039

[4] Dong XY Azzam MMM Zou XT. Effects of dietary threonine supplementation on intestinal barrier function and gut microbiota of laying hens. Poult. Sci. (2017) 96:3654–63. 10.3382/ps/pex185 28938780

[5] Potturi PV Patterson JA Applegate TJ. Effects of delayed placement on intestinal characteristics in turkey poults. Poult Sci (2005) 84:816–24. 10.1093/ps/84.5.816 15913196

[6] Burkholder KM Thompson KL Einstein ME Applegate TJ Patterson JA. Influence of stressors on normal intestinal microbiota, intestinal morphology, and susceptibility to *Salmonella enteritidis* colonization in broilers. Poult. Sci. (2008) 87:1734–41. 10.3382/ps.2008-00107 18753440

[7] Timbermont L Lanckriet A Dewulf J Nollet N Schwarzer K Haesebrouck F. Control of Clostridium perfringens-induced necrotic enteritis in broilers by target-released butyric acid, fatty acids and essential oils. Avian Pathol. (2010) 39:117–21. 10.1080/03079451003610586 20390546

[8] Tan J Applegate TJ Liu S Guo Y Eicher SD. Supplemental dietary L-Arginine attenuates intestinal mucosal disruption during a coccidial vaccine challenge in broiler chickens. Br J Nutr. (2014) 112:1098–109. 10.1017/S0007114514001846 25181320

[9] Quinteiro-Filho WM Ribeiro A Ferraz-de-Paula V Pinheiro ML Sakai M Sá LR. Heat stress impairs performance parameters, induces intestinal injury, and decreases macrophage activity in broiler chickens. Poult. Sci. (2010) 89:1905–14. 10.3382/ps.2010-00812 20709975

[10] Dekich MA. Broiler industry strategies for control of respiratory and enteric diseases. Poult Sci. (1998) 77:1176–80. 10.1093/ps/77.8.1176 9706085 PMC7107213

[11] Sklan D Shelly M Makovsky B Geyra A Klipper E Friedman A. The effect of chronic feeding of diacetoxyscirpenol and T-2 toxin on performance, health, small intestinal physiology and antibody production in turkey poults. Br Poult Sci. (2003) 44:46–52. 10.1080/0007166031000085373 12737225

[12] Thornton PK. Livestock production: recent trends, future prospects. Philos Trans R Soc Lond Ser B (2010) 365:2853–67. 10.1098/rstb.2010.0134 20713389 PMC2935116

[13] Windhorst HW Changes in poultry production and trade worldwide. Worlds Poult Sci. (2006) 62:585–602. 10.1017/S0043933906001140

[14] Frost AJ Antibiotics and animal production. In: Woolcock JB, editor. World's Animal Science Microbiology of Animals and Animal Products. (New York, NY: Elsevier (1991). p 181–94.

[15] Center for European Agricultural Studies (CEAS) The impact on animal husbandry in the European community of the use of growth promoters. Pages 1–319 in Growth Promoters in Anim. Feed. Rep. Eur. Comm. London Univ. Press, U. K. (1991).

[16] Coates ME Davies ME Kon SK. The effect of antibiotics on the intestine of the chick. Brit J Nutr. (1955) 9:110–9. 10.1079/BJN19550016 14351666

[17] Lev M Forbes M. Growth response to dietary penicillin of germ-free chicks and of chicks with a defined intestinal flora. Brit J Nutr. (1959) 13:78–84. 10.1079/BJN19590012 13628943

[18] Coates ME Fuller R Harrison GF Lev M Suffolk SF. Comparison of the growth of chicks in the Gustafsson germ-free apparatus and in a conventional environment, with and without dietary supplements of penicillin. Brit J Nutr. (1963) 17:141–50. 10.1079/BJN19630015 14021819

[19] Gaskins HR Collier CT Anderson DB. Antibiotics as growth promotants: Mode of action. Anim Biotechnol. (2002) 13:29–42. 10.1081/ABIO-120005768 12212942

[20] Dibner JJ Richards JD. Antibiotic growth promoters in agriculture: history and mode of action. Poult Sci. (2005) 84:634–43. 10.1093/ps/84.4.634 15844822

[21] van den Broek PJ. Antimicrobial drugs, microorganisms, and phagocytes. Rev. Infect. Dis. (1989) 11:213–45. 10.1093/clinids/11.2.213 2649959

[22] Woo PC Tsoi HW Wong LP Leung HC Yuen KY. Antibiotics modulate vaccine-induced humoral immune response. Clin Diagn Lab Immun. (1999) 6:832–7. 10548572 10.1128/cdli.6.6.832-837.1999 PMC95784

[23] Niewold TA. The nonantibiotic anti-inflammatory effect of antimicrobial growth promoters, the real mode of action? A hypothesis. Poult Sci. (2007) 86:605–9. 10.1093/ps/86.4.605 17369528

[24] Costa E Uwiera RR Kastelic JP Selinger LB Inglis GD. Non-therapeutic administration of a model antimicrobial growth promoter modulates intestinal immune responses. Gut Pathog. (2011) 3:14. 10.1186/1757-4749-3-14 21943280 PMC3195107

[25] Porter REJr. Bacterial enteritides of poultry. Poult Sci. (1998) 77:1159–65. 10.1093/ps/77.8.1159 9706083

[26] Dalloul RA Lillehoj HS. Poultry coccidiosis: recent advancements in control measures and vaccine development. Expert Rev Vaccines. (2006) 5:143–63. 10.1586/14760584.5.1.143 16451116

[27] Castanon JIR History of the use of antibiotics as growth promoters in European poultry feeds. Poult Sci. (2007) 86:2466–71. 10.3382/ps.2007-00249 17954599

[28] Lillehoj HS Lillehoj EP. Avian coccidiosis. A review of acquired intestinal immunity and vaccination strategies. Avian Dis. (2000) 44:408–25. 10.2307/1592556 10879922

[29] Lillehoj HS Trout JM. Coccidia: a review of recent advances on immunity and vaccine development. Avian Pathol. (1993) 22:3–31. 10.1080/03079459308418897 18670994

[30] Williams RB. Intercurrent coccidiosis and necrotic enteritis of chickens: Rational, integrated disease management by maintenance of gut integrity. Avian Pathol. (2005) 34:159–80. 10.1080/03079450500112195 16191699

[31] Matthews JO Southern LL. The effect of dietary betaine in *Eimeria acervulina*-infected chicks. Poult Sci. (2000) 79:60–5. 10.1093/ps/79.1.60 10685890

[32] Persia ME Young EL Utterback PL Parsons CM. Effects of dietary ingredients and *Eimeria acervulina* infection on chick performance, apparent metabolizable energy, and amino acid digestibility. Poult Sci. (2006) 85:48–55. 10.1093/ps/85.1.48 16493945

[33] Adedokun SA Helmbrecht A Applegate TJ. Investigation of the effect of coccidial vaccine challenge on apparent and standardized ileal amino acid digestibility in grower and finisher broilers and its evaluation in 21-day-old broilers. Poult Sci. (2016) 95:1825–35. 10.3382/ps/pew066 26957634

[34] Adedokun SA Adeola O The response in jejunal and ileal nutrient and energy digestibility and the expression of markers of intestinal inflammation in broiler chickens to coccidial vaccine challenge and phytase supplementation. Can J Anim Sci. (2017) 97:258–67. 10.1139/cjas-2016-0093

[35] Kim E Leung H Akhtar N Li J Barta JR Wang Y. Growth performance and gastrointestinal responses of broiler chickens fed corn-soybean meal diet without or with exogenous epidermal growth factor upon challenge with *Eimeria*. Poult Sci. (2017) 96:3676–86. 10.3382/ps/pex192 28938785 PMC5850350

[36] Thapa S Hinrichsenb LK Brenninkmeyer C Gunnarssond S Heerkens JLT Verwer C. Prevalence and magnitude of helminth infections in organic laying hens (*Gallus gallus* domesticus) across Europe. Vet Parasitol. (2015) 214:118–24. 10.1016/j.vetpar.2015.10.009 26518645

[37] Windisch W Kroismayr A The Effects of Phytobiotics on Performance and Gut Function in Monogastrics (2006). Available online at: https://en.engormix.com/feed-machinery/articles/phytobiotics-on-performance-gut-function-in-monogastrics-t33528.htm (Accessed September 10, 2018).

[38] Georgieva NV Gabrashanska M Koinarski V Yaneva Z. Zinc supplementation against *Eimeria acervulina*-induced oxidative damage in broiler chickens. Vet. Med. Int. (2011) 2011:647124. 10.4061/2011/647124 21461390 PMC3065002

[39] Gao T Zhao MM Zhang L Li JL Yu LL Lv PA Effect of in ovo feeding of L-arginine on the development of lymphoid organs and small intestinal immune barrier function in post-hatch broilers. Anim Feed Sci Tech. (2017) 225:8–19. 10.1016/j.anifeedsci.2017.01.004

[40] Bun SD Guo YM Guo FC Ji F Cao H. Influence of organic zinc supplementation on the antioxidant status and immune responses of broilers challenged with *Eimeria tenella*. Poult Sci. (2011) 90:1220–6. 10.3382/ps.2010-01308 21597062

[41] Hooper LV Gordon JI. Commensal host-bacterial relationships in the gut. Science (2001) 292:1115–8. 10.1126/science.1058709 11352068

[42] Choct M. Managing gut health through nutrition. Brit Poult Sci. (2009) 50:9–15. 10.1080/00071660802538632 19234925

[43] Rhee KJ Sethupathi P Driks A Lanning DK Knight KL. Role of commensal bacteria in development of gut-associated lymphoid tissues and preimmune antibody repertoire. J Immunol. (2004) 172:1118–24. 10.4049/jimmunol.172.2.1118 14707086

[44] Brisbin JT Gong J Sharif S. Interactions between commensal bacteria and the gut-associated immune system of the chicken. Anim Health Res Rev. (2008) 9:101–10. 10.1017/S146625230800145X 18541076

[45] Kasahara Y Chen CL Gobel TWF Bucy RP Cooper MD Intraepithelial lymphocytes in birds. In: Kiyono H McGhee JR, edotors. Mucosa Immunology: Intraepithelial Lymphocytes. New York, NY: Raven Press (1993). p. 163–74.

[46] Jeurissen SHM Vervelde L Janse EM Structure and function of lymphoid tissues of the chicken. Poult Sci Rev. (1994) 5:183–207.

[47] Bar-Shira E Sklan D Friedman A. Establishment of immune competence in the avian GALT during the immediate post-hatch period. Dev Comp Immunol. (2003) 27:147–57. 10.1016/S0145-305X(02)00076-9 12543128

[48] Klasing KC. Nutritional modulation of resistance to infectious diseases. Poult Sci. (1998) 77:1119–25. 10.1093/ps/77.8.1119 9706075

[49] Lin H Jiao HC Buyse J Decuypere E Strategies for preventing heat stress in poultry. World Poult Sci. (2006) 62:71–86. 10.1079/WPS200585

[50] Alleman F Leclercq B. Effect of dietary protein and environmental temperature on growth performance and water consumption of male broiler chickens. Brit Poult Sci. (1997) 38:607–10. 10.1080/00071669708418044 9511009

[51] Temim S Chagneau AM Peresson R Tesseraud S Chronic heat exposure alters protein turnover of three different skeletal muscles in finishing broiler chickens fed 20 or 25% protein diets. J Nutr. (2000) 130:813–9. 10.1093/jn/130.4.813 10736335

[52] Field CJ Johnson IR Schley PD. Nutrients and their role in host resistance to infection. J Leukocyte Biol. (2002) 71:6–32. 10.1189/jlb.71.1.16 11781377

[53] Calder PC Yaqoob P Amino acids and immune function. In: Cynober LA, editor. Metabolic and Therapeutic Aspects of Amino Acids in Clinical Nutrition, 2nd ed. Boca Raton, FL: CRC Press (2004). p. 305–20.

[54] Ruth MR Field CJ. The immune modifying effects of amino acids on gut-associated lymphoid tissue. J Anim Sci Biotechnol. (2013) 4:1–10. 10.1186/2049-1891-4-27 23899038 PMC3750756

[55] Wu G Flynn NE Flynn SP Jolly CA Davis PK. Dietary protein or arginine deficiency impairs constitutive and inducible nitric oxide synthesis by young rats. J. Nutr. (1999). 129:1347–54. 10395597 10.1093/jn/129.7.1347

[56] Li P Yin YL Li D Kim SW Wu G. Amino acids and immune function. Br. Jour. Nutr. (2007) 98:237–52. 10.1017/S000711450769936X 17403271

[57] Kidd MT. Nutritional modulation of immune function in broilers. Poult Sci. (2004) 83:650–7. 10.1093/ps/83.4.650 15109062

[58] Payne CJ Scott TR Dick JW Glick B. Immunity to *Pasteurella multocida* in protein-deficient chickens. Poult Sci. (1990) 69:2134–42. 10.3382/ps.0692134 2084673

[59] Kim SW Mateo RD Yin YL Wu G Functional amino acids and fatty acids for enhancing production performance of sows and piglets. Asian Austral J Anim Sci. (2007) 20:295–306. 10.5713/ajas.2007.295

[60] Le Floc'h N Melchior D Obled C Modifications of protein and amino acid metabolism during inflammation and immune system activation. Livest. Prod. Sci. (2004) 87:37–45. 10.1016/j.livprodsci.2003.09.005

[61] Tan J Guo Y Applegate DE Zhao X L-Arginine regulates immune functions in chickens immunized with intermediate strain of infectious bursal disease vaccine. Jpn Poult Sci. (2015) 52:101–8. 10.2141/jpsa.0140101

[62] Lee JE Austic RE Naqi SA Golemboski KA Dietert RR. Dietary arginine intake alters avian leukocyte population distribution during infectious bronchitis challenge. Poult Sci. (2002) 81:793–8. 10.1093/ps/81.6.793 12079045

[63] Ban H Shigemitsu K Yamatsuji T Haisa M Nakajo T Takaoka M. Arginine and leucine regulate p70 S6 kinase and 4E-BP1 in intestinal epithelial cells. Int J Mol Med. (2004) 13:537–43. 10.3892/ijmm.13.4.537 15010853

[64] Andrews F.J Griffiths R. D. (2002). Glutamine: Essential for immune nutrition in the critically ill. Br. J. Nutr. 87, 3–8 10.1079/BJN2001451 11895153

[65] Watford M Glutamine and glutamate: Nonessential or essential amino acids? Anim Nutr. (2015) 1:119–22. 10.1016/j.aninu.2015.08.008 29767158 PMC5945979

[66] Newsholme P Why is L-glutamine metabolism important to cells of the immune system in health, post injury, surgery or infection? J Nutr. (2001) 131:515S−2522S. 10.1093/jn/131.9.2515S 11533304

[67] Dai SF Wang LK Wen AY Wang LX Jin GM Dietary glutamine supplementation improves growth performance, meat quality and color stability of broilers under heat stress. Br Poult Sci. (2009) 50:333–40. 10.1080/00071660902806947 19637033

[68] Bartell SM Batal AB. The effect of supplemental glutamine on growth performance, development of the gastrointestinal tract, and humoral immune response of broilers. Poult Sci. (2007) 86:1940–7. 10.1093/ps/86.9.1940 17704382

[69] Wu GY Field CJ Marliss EB. Glutamine and glucose metabolism in rat splenocytes and mesenteric lymph node lymphocytes. Am J Physiol Endoc M (1991) 260:E141–7. 1670976 10.1152/ajpendo.1991.260.1.E141

[70] Parry-Billings M Calder PC Newsholme EA Evans J. Does glutamine contribute to immunosuppression after major burns? Lancet (1990) 336:523–5. 10.1016/0140-6736(90)92083-T 1975037

[71] Horn NL Donkin SS Applegate TJ Adeola O. Intestinal mucin dynamics: Response of broiler chicks and White Pekin ducklings to dietary threonine. Poult Sci. (2009) 88:1906–14. 10.3382/ps.2009-00009 19687276

[72] Hegedus M Bokori J Andrasofszky E. The value of crude protein content and *in vitro* pepsin digestibility of abattoir by-product meals in the prediction of their available protein content. Acta Vet Hung. (1989) 37:27–33. 2516710

[73] Ravindran V Hendriks WH Camden BJ Thomas DV Morel PCH Butts CA Amino acid digestibility of meat and bone meals for broiler chicken. Aust J Agr Res. (2002) 53:1257–64. 10.1071/AR02055

[74] Garcia RA Phillips JG Physical distribution and characteristics of meat and bone meal protein. J. Sci. Food Agr. (2009) 89:329–36. 10.1002/jsfa.3453

[75] Barrier-Guillot B Jondreville ZC Changneau AM Larbier M Leuillet M Effect of heat drying temperature on the nutritive value of corn in chickens and pigs. Anim Feed Sci Tech. (1993) 41:149–59. 10.1016/0377-8401(93)90120-9

[76] Fisher DK Thompson DB Retrogradation of maize starch after thermal treatment within and above the gelatinization temperature range. Cereal Chem. (1997) 74:344–51. 10.1094/CCHEM.1997.74.3.344

[77] O'Brian S Wang YJ Susceptibility of annealed starches to hydrolysis by α-amylase and glucoamylase. Carbohyd Polym. (2007) 72:597–607. 10.1016/j.carbpol.2007.09.032

[78] Moran ETJr. Intestinal events and nutritional dynamics predispose *Clostridium perfringens* virulence in broilers. Poult Sci. (2014) 93:3028–36. 10.3382/ps.2014-04313 25260526

[79] Cowieson AJ Strategic selection of exogenous enzymes for corn/soy-based poultry diets. The J Poult Sci. (2010) 47:1–7. 10.2141/jpsa.009045

[80] Paris NE Wong EA. Expression of digestive enzymes and nutrient transporters in the intestine of *Eimeria maxima*-infected chickens. Poult Sci. (2013) 92:1331–5. 10.3382/ps.2012-02966 23571343

[81] Wu G Bazer FW Dai Z Li D Wang J Wu Z. Amino acid nutrition in animals: protein synthesis and beyond. Annu Rev Anim Biosci. (2014) 2:387–417. 10.1146/annurev-animal-022513-114113 25384149

[82] Moran ETJr. Digestion and absorption of carbohydrates in fowl and events through perinatal development. J Nutr. (1985) 115:665–74. 10.1093/jn/115.5.665 2582103

[83] Friedman A Sklan D. Impaired T lymphocyte immune response in vitamin A depleted rats and chicks. Br J Nutr. (1989) 62:439–49. 10.1079/BJN19890044 2479411

[84] Lessard M Hutchings D Cave NA. Cell-mediated and humoral immune responses in broiler chickens maintained on diets containing different levels of vitamin A. Poult Sci. (1997) 76:1368–78. 10.1093/ps/76.10.1368 9316112

[85] Friedman A Meidovsky A Leitner G Sklan D Decreased resistance and immune response to *Escherichia coli* infection in chicks with low or high intakes of vitamin A. J Nutr. (1991) 121:395–400. 2002410 10.1093/jn/121.3.395

[86] Cook ME Nutrition and immune response to the domestic fowl. Crit Rev Poult Biol. (1991) 3:167–89.

[87] Erf GF Bottje WG Bersi TK Headrick MD Fritts CA. Effects of dietary vitamin E on the immune system in broilers: altered proportions of CD4 T cells in the thymus and spleen. Poult Sci. (1998) 77:529–37. 10.1093/ps/77.4.529 9565234

[88] Ferket PR Qureshi MA. Performance and immunity of heat-stressed broilers fed vitamin- and electrolyte supplemented drinking water. Poult Sci. (1992) 71:88–97. 10.3382/ps.0710088 1539026

[89] Colnago GL Jensen LS Long PL. Effect of selenium and vitamin E on the development of immunity to coccidiosis in chickens. Poult Sci. (1984) 63:1136—3. 6739404 10.3382/ps.0631136

[90] Yazdankhah S Rudi K Bernhoft A. Zinc and copper in animal feed–development of resistance and co-resistance to antimicrobial agents in bacteria of animal origin. Microb Ecol Health Dis. (2014) 25:25862. 10.3402/mehd.v25.25862 25317117 PMC4179321

[91] Gielda LM DiRita VJ. Zinc competition among the intestinal microbiota. M Bio. (2012) 3:e00171–12. 10.1128/mBio.00171-12 22851657 PMC3419517

[92] Adeola O Cowieson AJ. Board invited review: opportunities and challenges in using exogenous enzymes to improve nonruminant animal production. J Anim Sci. (2011) 89:3189–218. 10.2527/jas.2010-3715 21512114

[93] Bedford MR Removal of antibiotic growth promoters from poultry diets: implications and strategies to minimize subsequent problems. World Poult Sci. (2000) 56:347–65. 10.1079/WPS20000024

[94] Jia W Slominski BA Bruce HL Blank G Crow G Jones O. Effects of diet type and enzyme addition on growth performance and gut health of broiler chickens during subclinical *Clostridium perfringens* challenge. Poult Sci. (2009) 88:132–40. 10.3382/ps.2008-00204 19096067

[95] De Silva SD Hesselman K Åman P Effects of water and [beta]-glucanase treatment on non-starch polysaccharides in endosperm of low and high viscous barley. Swedish J Agric Res. (1983) 13:211–9.

[96] Gibson GR Roberfroid MB. Dietary modulation of the human colonic microbiota: introducing the concept of prebiotics. J Nutr. (1995) 125:1401–12. 7782892 10.1093/jn/125.6.1401

[97] Bohak Z. Purification and characterization of chicken pepsinogen and chicken pepsin. J Biol Chem. (1969) 244:4638–48. 4897245

[98] Kaufman HW Kleinberg I. The effect of pH on calcium binding by phytic acid and its inositol phosphoric acid derivatives and on the solubility of their calcium salts. Arch Oral Biol. (1971) 16:445–60. 10.1016/0003-9969(71)90168-3 5281215

[99] Crea F Crea P De Robertis A Sammartano S Speciation of phytate ion in aqueous solution. Characterization of Ca-phytate sparingly soluble species. Chem Spec Bioavailab. (2004) 16:53–9. 10.3184/095422904782775090

[100] Cuppett SL Hall CC. Antioxidant activity of *Labiatae*. Adv Food Nutr Res. (1998) 42:245–71. 10.1016/S1043-4526(08)60097-2 9597729

[101] Wei A Shibamoto T. Antioxidant Activities and volatile constituents of various essential oils. J Agric Food Chem. (2007) 55:737–1742. 10.1021/jf062959x 17295511

[102] Chrubasik S Pittler MH Roufogalis BD Zingiberis rhizome: A comprehensive review on the ginger effect and efficacy profiles. Phytomedicine (2005) 12:684–701. 10.1016/j.phymed.2004.07.009 16194058

[103] Windisch W Schedle K Plitzner C Kroismaysch A. Use of phyogenic products as feed additives for swine and poultry. J Anim Sci. (2008) 86:140–8. 10.2527/jas.2007-0459 18073277

[104] Wang R Li D Bourne S Can 2000 years of herbal medicine history help us solve problems in the year 2000. In: Proceedings of Alltech's 14th Annual Symposium (AAS'98). Kentucky (1998). p 273–291.

[105] Celi P Cowieson AJ Fru-Nji F Steinert RE Kluenter AM Verlhac V Gastrointestinal functionality in animal nutrition and health: new opportunities for sustainable animal production. Anim Feed Sci Tech. (2017) 234:88–100. 10.1016/j.anifeedsci.2017.09.012

